# A pilot study of a Community Health Agent-led type 2 diabetes self-management program using Motivational Interviewing-based approaches in a public primary care center in São Paulo, Brazil

**DOI:** 10.1186/s12913-016-1968-3

**Published:** 2017-01-13

**Authors:** Thais Moura Ribeiro do Valle Nascimento, Ken Resnicow, Marcia Nery, Alexandra Brentani, Elizabeth Kaselitz, Pooja Agrawal, Simanjit Mand, Michele Heisler

**Affiliations:** 1 Division of Endocrinology, Department of Internal Medicine, Faculdade de Medicina da Universidade de São Paulo, FMSUP--Medical Faculty of the University of São Paulo, São Paulo, SP Brazil; 2 Unidade Básica de Saúde (UBS—Basic Health Unit) Vila Piauí, Fundacão Faculdade de Medicina da FMUSP, Western Region of São Paulo, SP Brazil; 3 Department of Health Behavior and Health Education, School of Public Health, University of Michigan, Ann Arbor, MI USA; 4 Department of Pediatrics, University of São Paulo Medical School, São Paulo, Brazil; 5 University of Michigan Medical School, Ann Arbor, MI USA; 6 Department of Internal Medicine, University of Michigan Medical School, Ann Arbor, MI USA; 7 Center for Clinical Management Research, Ann Arbor Veterans’ Affairs (VA) Healthcare System, 1600 Plymouth Drive, Building 16, Ann Arbor, MI 48109 USA

**Keywords:** Diabetes, Community health worker, Pilot study, Self-management, Motivational Interviewing, Brazil, Primary care

## Abstract

**Background:**

Rates of noncommunicable diseases (NCDs) such as type 2 diabetes are escalating in low and middle-income countries such as Brazil. Scalable primary care-based interventions are needed to improve self-management and clinical outcomes of adults with diabetes. This pilot study examines the feasibility, acceptability, and outcomes of training community health agents (CHAs) in Motivational Interviewing (MI)-based counseling for patients with poorly controlled diabetes in a primary care center in São Paulo, Brazil.

**Methods:**

Nineteen salaried CHAs participated in 32 h of training in MI and behavioral action planning. With support from booster training sessions, they used these skills in their regular monthly home visits over a 6 month period with 57 diabetes patients with baseline HbA1cs > 7.0%. The primary outcome was patients’ reports of the quality of diabetes care as measured by the Portuguese version of the Patient Assessment of Chronic Illness Care (PACIC) scale. Secondary outcomes included changes in patients’ reported diabetes self-management behaviors and in A1c, blood pressure, cholesterol and triglycerides. We also examined CHAs’ fidelity to and experiences with the intervention.

**Results:**

Patients reported improvements over the 6 month period in quality of diabetes care received (PACIC score improved 33 (+/−19) to 68 (+/−21) (*p* < .001)). They reported increases in physical activity (*p* = .001), consumption of fruits and vegetables (*p* < .001) and medication adherence (*p* = .002), but no decreases in consumption of high-fat foods (*p* = .402) or sweets (*p* = .436). Participants had mean 6-month A1c levels 0.34% points lower than at baseline (*p* = .08) and improved mean LDL (−16.1 mg/dL, *p* = .005) and triglyceride levels (−38.725 mg/dL, *p* = .002). Of the 16 CHAs observed in fidelity assessments, 13 were categorized as medium- or high-performing on MI skills, while 3 were low-performing. CHAs expressed enthusiasm about learning new skills, and many described a shift from advice-giving to encouraging patients to define their own goals.

**Conclusion:**

In resource-scarce settings, it is essential to fully utilize existing primary care resources to stem the epidemic of diabetes and other NCDs. Our pilot results support the potential of training CHAs to incorporate effective diabetes self-management support into their routine patient encounters.

**Trial registration:**

NCT02994095 12/14/2016 Registered retrospectively.

## Background

While the increase in noncommunicable diseases (NCD) is a global phenomenon, low and middle income countries (LMICs) carry a disproportionate burden. Nearly 80% of NCD-related deaths occur in LMICs [1] with a younger age of disease onset than in high income countries, leading to a greater loss of healthy years and economic productivity [2]. This rise in NCDs is attributed to a number of factors, including more sedentary lifestyles, a transition to processed foods often high in fat, salt and sugar [3], and a dramatic increase in obesity rates [4]. In Latin America an estimated 1.6 million people die from NCDs annually and many prematurely, with nearly a half million dying before the age of 70 [5, 6]. In Brazil, in 2013 NCDs accounted for 73% of deaths [6]. Unfortunately, there is a paucity of evidence on cost-effective interventions to address this disease burden [7].

Type 2 diabetes (T2DM) is one of the most prevalent NCDs worldwide [8]. The number of adults with T2DM has quadrupled worldwide in four decades to 422 million. Worldwide, diabetes complications account for over 2 million deaths a year [9], are the seventh leading cause of disability and lead to direct annual costs of $825 billion [9, 10]. In 2012, it was estimated that 4 of 5 patients with diabetes resided in LMICs [11, 12]. Brazil has the 4th highest prevalence of T2DM in the world, with 11.9 million (or roughly 10%) of Brazilians with diabetes [8, 13]. In 2010 diabetes was estimated to be responsible for 278,778 years of potential life lost for every million people in Brazil. In 2000 the estimated annual direct cost of diabetes was USD $4.95 billion. As in other countries, T2DM rather than type 1 DM (which accounts for approximately 5% of cases of diabetes in Brazil) constitutes the bulk of this morbidity, mortality, and costs. Moreover, although there is no systematic screening for prediabetes, it is estimated that prevalence of prediabetes in Brazil is high and increasing rapidly [14]. Simōes et al. describe programs implemented by the Brazilian Ministry of Health to address this diabetes epidemic, but there is not yet evidence on the effectiveness of these interventions. Success will depend largely on the development of scalable interventions that enhance patients’ ability to effectively manage their diabetes and to make and sustain healthy behavior changes (“self-management”) [15].

The Brazil Family Health Strategy (FHS) provides a unique opportunity to develop and test innovative primary care-based interventions to improve health behaviors and outcomes. Using a community-based approach to administering primary care, the FHS deploys within each primary care health center (Basic Health Unit, or *Unidade Básica de Sáude*, UBS) multidisciplinary health teams typically consisting of a physician, a nurse, two nurse assistants, and four to six salaried community health agents (CHAs) assigned to specific geographic catchment areas [16]. The CHAs provide a link between families and primary health care centers by visiting patients’ homes monthly and gathering data on patients’ health and social conditions and providing support between clinic visits with other health team members. There is a growing body of evidence that CHA-led programs improve health outcomes in a range of settings and for multiple health conditions [17–22], including for diabetes [23–29]. A recent review emphasizes the importance of focusing on primary care and the potential of deploying CHAs already employed in primary care clinics to reduce the NCD burden in LMICs [11].

Yet, CHAs employed by primary care centers have multiple responsibilities and face multiple demands on their time. Moreover, they generally do not receive formal or rigorous training in behavioral counseling techniques such as Motivational Interviewing [30] to guide their outreach to adults with diabetes and other NCDs that require high levels of patient self-management to improve outcomes. There is strong evidence on the effectiveness of Motivational Interviewing-based brief counseling to improve healthy behaviors and outcomes among adults with type 2 diabetes [31, 32]. Yet, to date there is little rigorous evidence on the feasibility, acceptability, and effectiveness of training CHAs in Brazil’s Basic Health Units (UBSs) in these evidence-based behavioral counseling skills for chronic disease self-management.

To address this lack of evidence, we tested a model for CHAs to provide effective diabetes self-management support in their monthly home visits with adults with poor glycemic control. We developed and evaluated a pilot study of a diabetes self-management support intervention led by the salaried CHAs at one primary care center (UBS) serving low-income communities in the western region of the city of São Paulo. We examined the feasibility and acceptability of training the CHAs in Motivational-Interviewing (MI) based communication approaches and action planning to support diabetes self-management. We then tracked clinical and self-reported outcomes among adult patients with poor glycemic control who were assigned to these CHAs. The study underwent Institutional Review Board review and was approved by the participating institutions.

## Methods

### Setting

This study was implemented in a Primary Care Health Unit (UBS- Unidade Básica de Saúde) located in a low-income community in the western region of the city of Sao Paulo. As of early 2015, 13,000 people received care at the center, with 713 adults with type 2 diabetes. Care at the UBS is organized along the lines of Brazil’s Family Health Strategy (FHS), with 4 health care teams each composed of one doctor, one nurse, two nursing assistants, and six community health agents (CHAs). Each team covers specific neighborhoods within the served communities. The CHAs are recruited among residents from those communities. No formal training is required besides having some secondary education. As in other UBSs, the CHAs receive an initial 40 h of training in their work duties over their first weeks of work that does not include training in behavioral counseling. During this time, they learn much about their roles and responsibilities by shadowing other CHAs when they are first hired. Each CHA is assigned approximately 150 families that they are expected to visit at least once a month.

### Recruitment of patients

Potentially eligible participants with type 2 diabetes were identified from medical records. Participants needed to have an A1c of >7.0% in the prior 6 months and again at the time of recruitment for the intervention to be eligible. Exclusion criteria were age more than 75 years old (as recommended glycemic targets are higher for adults in this age group), being pregnant, terminal health conditions, and conditions (e.g., severe mental illness, dementia) that would impede meaningful participation. Participants who agreed to participate in the study completed a baseline survey and a clinical assessment in which blood pressure was measured and blood was drawn to be sent for analyses of A1c and cholesterol levels at the Central Laboratory of the Clinic Hospital of the University of São Paulo. A1c was measured using standard internationally validated turbidimetric inhibition immunoassay methods (Roche Cobas C11).

### Description of the intervention

#### Participating CHA characteristics

Nineteen of the UBS’s 24 community health agents (CHAs) were trained and delivered the intervention. All the CHAs were women between the ages of 25 and 60 with a mean age of 47. Forty seven percent of CHAs completed high school, while 37% had less than a high school degree and 15% had begun or completed college. They had spent an average of 34 years in the community and had worked as CHAs for an average of 7 years, with a range of 1 to 13 years. Nearly 90% of participating CHAs had at least one family member living in the community at the time of the intervention.

#### CHA training

Before the intervention period began in March, 2014, 19 of the UBS’s salaried CHAs participated in 32 h of initial training in MI led by Brazilian trainers who had themselves been trained by research team members (MH, KR). The training focused on autonomy-supportive MI communication skills to help adults with diabetes: 1) identify their own diabetes self-management goals (e.g., taking medications, increasing physical activity, eating healthier, etc.) and then 2) formulate specific short-term small steps (“action plans”) to incorporate these healthier behaviors in their daily lives. In addition, CHAs participated in 4 h a month of booster training and support over the course of the 6-month intervention period.

MI is an evidence-based counseling approach to help motivate patients to change unhealthy behaviors and adhere to treatment recommendations [33]. When delivered by trained health professionals, MI has been found to lead to healthy behavior changes across a variety of health behaviors [34] and health conditions [35, 36], including diabetes [37–41]. Although there is less evidence on use of MI by lay health workers such as CHAs, in several prior interventions in the United States we and other researchers have developed training approaches for community health workers and paraprofessionals that led to improved health behaviors and outcomes among adults with diabetes and other conditions [28, 42–45]. These approaches and materials were adapted for use in this intervention.

#### Outcome measures

Self-report outcomes were measured via survey at baseline and 6 months after baseline. Our primary outcome was participants’ assessment of the quality of the diabetes care they received from their UBS health care team, measured with the validated Portuguese version [46] of the Patient Assessment of Chronic Illness Care (PACIC) [47, 48], in particular the sub-scales of goal-setting, problem-solving, and quality of follow-up care to clinic visits. In order to assess changes in diabetes self-management behaviors targeted in CHAs’ efforts to help participants make action plans, our second outcomes were changes in healthy eating (fruits, vegetables, fats and sweets consumption), and physical activity as measured by the Portuguese version [49] of the Summary of Diabetes Self-Care Activities (SDSCA) [50], and adherence to medications as measured by the Portuguese version [51] of the Morisky measure [52]. To assess CHA counseling skills, participants completed items from the Health Care Climate Questionnaire (HCCQ) that were translated and back-translated into Portuguese [53, 54]. To facilitate comparison across the measures, all outcome measures except the Morisky adherence measure were scored from 0 to 100, with higher scores indicating more positive assessments. The 4-item Morisky scale was scored from 0 to 4, with higher scores indicating worse adherence (0- high adherence, 1–2 medium adherence, 3–4 low adherence). Clinical measures were pulled from participants’ medical records at baseline and 6 months after baseline. Measures included HbA1c, blood pressure, cholesterol and triglycerides.

#### CHA fidelity checklist

Research team members directly observed a random sample of CHAs’ home visits with study participants and rated their motivational interviewing skills with a fidelity checklist adapted from the “1-Pass Coding System for Motivational Interviewing” [55]. The checklist originally had 47 items, with each item being scored on a 7-point scale, with 7 signifying complete adherence to the evaluated skill. Measures included: reviewing previous action plans; identifying barriers and brainstorming solutions; providing encouragement and supporting patient autonomy; reflecting on patient values, concerns, and questions; identifying a manageable, important goal and creating a subsequent action plan; and creating a collaborative and supportive environment.

Sixteen of the 19 CHAs were evaluated in 31 home visits. Observations noting the presence and quality of target MI behaviors and actions were recorded during the interaction using the adapted checklist. Narrative comments assessing the overall quality of the CHA’s interactions with the participant were also made during each CHA encounter. The team then selected 10 MI techniques especially salient for a successful patient-CHA interaction (respecting patient talking time, expressing empathy, summarizing concerns for health team, supporting patient autonomy, focusing on patient values, offering action plan options, asking open-ended questions, providing positive reinforcement, not making judgmental statements). Each CHA was given an average score based on performance across all 10 selected traits and were stratified into three levels of performance (high performance defined as an average of 6–7, medium as 4–5, low as 1–3). We regarded the medium and high performing groups as CHAs that had successfully incorporated learned MI skills into their patient interactions.

#### Statistical analysis

We followed international guidelines for analysis and reporting of clinical trials [56] and used two-sided tests for all equality tests. STATA 13 was used for all analyses [57]. We used descriptive statistics (mean and percent) to summarize patient baseline characteristics including the baseline values on all our outcomes measures as well as socio-demographic characteristics (age, gender, education), health literacy, self-rated health, duration of diabetes, whether participants were on insulin, and number of oral anti-hyperglycemic medication. We assessed significant differences in mean values between baseline and 6 month for our primary outcome of the PACIC, for the secondary outcomes of three self-management behaviors, and in the measured clinical measures using paired t-tests for continuous measures. To assess patients’ experience with their CHAs as measured by the HCCQ items, we examined percent of participants who chose “agree” or “totally agree” for each item. For the four HCCQ items measured at both baseline and 6-month follow-up, we tested the equality of proportions between the two periods [57].

## Results

### Participant flow and baseline data

**Fig. 1:**
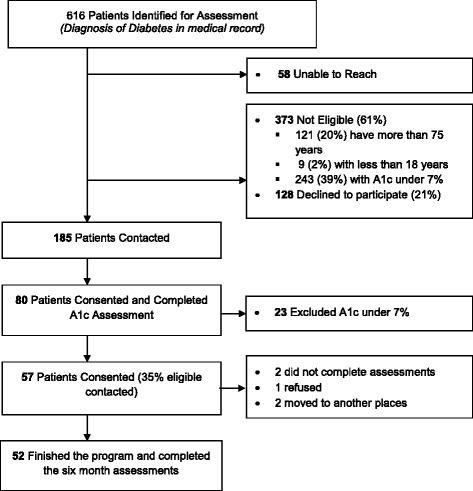
CONSORT flow diagram

Figure 1 shows participant flow. Of 150 contacted patients, 83 (55%) agreed to participate. 57 of these continued to have A1c levels > 7% in the baseline lab assessment and were enrolled in the study. Participants’ baseline characteristics are reported in Table 1. Five of the 57 study participants were lost to follow-up.

**Table 1:** Participant baseline characteristics (*N* = 57)

	Baseline
Age, mean years (SD)	62 (10)
Female, n (%)	58%
Years of formal education	
5 years or less	61%
6 to 13 years	32%
13 years or more	47%
Health literacy, n (%)	
Needs help reading forms (always or sometimes)	51%
Needs help writing down blood glucose numbers (always or sometimes)	31%
Self-rated Health, n (%)	
Very good to excellent	0%
Good	71%
Poor to Fair	28%
Duration of diabetes in years, mean (+/− SD)	14 (7.7)
Hemoglobin A1c, mean (SD)	8.8 (1.4)
Blood pressure, mean (SD)	
Systolic	141.0 (27.1)
Diastolic	77.6 (15.1)
LDL Cholesterol, mean (SD)	109.6 (39.1)
HDL Cholesterol, mean (SD)	43.7 (11.9)
Triglycerides, mean (SD)	185.7 (103.2)
BMI (kg/m2), mean (SD)	30.3 (5.9)
On insulin, n (%)	37 (65%)
Number of oral anti-hyperglycemic medications, n (%)	
0	8 (14%)
1	21 (37%)
2 or 3	28 (49%)

### Six month primary and secondary outcomes

As Table 2 shows, participants’ mean scores on the PACIC improved from 33 (+/−19) to 68 (+/−21) (*p* < .001), with improvements in the subscales of satisfaction with support for their goal-setting, problem-solving, and follow-up support between clinic visits (all *p* < .001). For our secondary outcomes of improvements in specific diabetes self-management behaviors, participants reported significant increases in consumption of fruits and vegetables (*p* < .001), in physical activity (*p* = .001), and in diabetes medication adherence (*p* = .002). There were no significant decreases in consumption of high-fat foods (*p* = .402) or in sweets (*p* = .436).

**Table 2:** Scores for intervention group across study measures

Primary Outcome	*N*	Mean baseline score^a^	Mean 6-month score	*p* - value
Overall PACIC Score, mean (SD)	43	33 (19)	68 (21)	*p* < .001
Goal-setting PACIC subscale, mean (SD)	52	29 (25)	71 (24)	*p* < .001
Problem-solving PACIC subscale, mean (SD)	52	13 (25)	63 (30)	*p* < .001
Follow up PACIC subscale, mean (SD)	43	29 (19)	56 (28)	*p* < .001
Exploratory Outcomes				
Number of Fruit/Vegetable Portions a Day (SD)	52	47 (22)	59 (20)	*p* < .001
Number of Days Had Five or More Fruit/vegetable (SD)	52	24 (42)	51 (40)	*p* < .001
Number of Days Ate High fat foods (SD)	52	32 (37)	37 (33)	*p* = .402
Number of Days Ate Sweets (SD)	52	80 (20)	77 (24)	*p* = .436
Number of Days 30 min of Exercise (SD)	52	21 (35)	39 (31)	*p* = .002
Diabetes Medication Adherence (SD)	52	2.98 (1.06)	2.40 (1.01)	*p* = .002

^**a**^All scores were on a 0–100 scale with higher values meaning more positive outcomes, except for the Morisky scale used to measure diabetes medication adherence that was scored on a 0–4 scale with higher values indicating worse reported adherence

### Respondents’ assessment of interactions with their CHAs

Table 3 shows respondent responses to individual items of the HCCQ assessing key dimensions of the CHAs’ interactions with them. There were statistically significant improvements in three of the four items asked at baseline and 6-month follow up (*p* < =.01 for all three) and marginal statistical improvement (*p* = .051) in the fourth (“My CHA encouraged me to ask questions about my diabetes.”). At 6 months 63% of respondents reported that their “relationship with [their] CHA improved significantly over the past 6 months.” 65% reported that their “diabetes care improved significantly over the past 6 months”.

**Table 3:** Participants’ Experiences with Their Community Health Agent (CHA)^a^

Item	% Participants Who ‘Agree’ or ‘Totally Agree’				
	Baseline (*n* = 56)	6 month (*n* = 52)	% 6 month—% Baseline^b^	95%CI	*p* - value
My CHA understood my point of view about my diabetes	73%	92%	19%	5.4–32.8%	0.009
My CHA expressed confidence in my ability to make decisions about my diabetes	64%	88%	24%	8.9–39.4%	0.003
My CHA understood how I want to take care of my diabetes	68%	88%	21%	5.6–35.6%	0.010
My CHA encouraged me to ask questions about my diabetes	57%	75%	18%	0.3–35.4%	0.051
I feel my CHA gave me options about how to control my diabetes	NA^c^	62%	-	-	-
My CHA helped me define specific goals for my diabetes	NA	71%	-	-	-
My CHA helped me resolve problems that came up with taking care of my diabetes	NA	50%	-	-	-
At times I felt negatively judged by my CHA	NA	12%	-	-	-
My CHA helped me identify my personal values that are important for me	NA	71%	-	-	-
My relationship with my CHA improved significantly over the past 6 months	NA	63%	-	-	-
My diabetes care improved significantly over the past 6 months	-	65%	-	-	-

^a^Items from Health Care Climate Questionnaire (HCCQ) with four response choices of “Totally Disagree,” “Disagree”, “Agree”, and “Totally Agree”

^b^Difference in proportion of Agree or Strongly Agree between baseline and 6 month values

^c^NA (Not assessed): These items were only asked at 6-month follow-up

### Changes in clinical values

Table 4 shows results of analyses examining changes in diabetes-relevant clinical values between baseline and 6 months for the 52 participants for whom we had baseline and 6-month follow up data. These participants had mean 6-month A1c levels 0.34% points lower than at baseline (*p* = .08). Respondents also had improved mean LDL levels (−16.1 mg/dL, *p* = .005) and triglyceride levels (−38.725 mg/dL, *p* = .002).

**Table 4:** Changes in clinical values between baseline and 6-month follow-up (*N* = 52)

	Baseline	6 month	6 month- baseline	*P* - value
Hemoglobin A1c, mean (SD)	8.8 (1.4)	8.4 (1.4)	−0.338	*p* = .082
Blood pressure, mean (SD)				
Systolic	143.0 (26.9)	146.9 (24.5)	3.923	*p* = .302
Diastolic	77.9 (15.2)	77.5 (11.7)	−0.404	*p* = .852
LDL Cholesterol, mean (SD)	107.9 (40.4)	91.8 (37.8)	−16.096	*p* = .005
HDL Cholesterol, mean (SD)	43.9 (12.3)	45.2 (12.5)	1.250	*p* = .119
Triglycerides, mean (SD)	186.3 (104.9)	147.6 (79.4)	−38.725	*p* = .002

### Fidelity Assessments of CHAs in home visits

The fidelity assessments found that key MI techniques were adopted by almost all of the CHAs, especially in the areas of supporting patient autonomy, expressing empathy and demonstrating a genuine concern for their patients. CHAs overall had weakest performance in avoiding judgment, focusing on patient values and summarizing health concerns to be conveyed to the health care team. Of the 16 CHAs observed, 13 were categorized as medium or high performing, while 3 CHAs were low-performing.

### Experiences of CHAs with the intervention

CHAs were interviewed to gain an in-depth understanding of their experience with this project, including the influence that they felt MI training had on their interactions with patients. Two major themes identified were: 1) the training led CHAs to shift from advice-giving to listening and question-asking; and 2) for the first time they started to give patients the opportunity to identify what they wanted to change and how in regards to their health behaviors. Many CHAs emphasized their new recognition of the importance of allowing patients to decide what is important to them without the imposition of the CHA’s own opinions or beliefs (“the patient is the master of his life and health. What is important to me cannot be the same for him. It comes from him.”). This shifts the role of the CHAs from a lecturer to an active listener, which is a key element of MI. This was summarized by a CHA who stated, “The training changed how we approach the person. Not to impose, but to question. [It] changed the way we talk: A new approach”. A CHA laughingly described CHAs as having “a famous reputation to give advice” and explained that this project showed her that this could change. Other remarks demonstrating a shift toward the patient-centered, patient-empowering approach of MI include: “[It] is not my time. [It] is the person’s time, which we must respect” and “when a person is empowered, they respond better”. One CHA said that she changed to “a ‘how can I help you,’ or ‘please help me understand’ approach”.

Another theme identified was that CHAs were excited to learn new methods to inform their practice and enhance their skills. When asked how the project changed how she functions at work, one CHA said, “[I] did even better. When they bring new methods, we have a motivation to work. Now we have different questions.” Other CHAs echoed this interest in learning new skills by describing the training as providing “a new vision”, “innovations that bring to light new methods” and “an opportunity to be acting in an organized manner”.

The vast majority of CHAs were positive about their experience. A few CHAs, however, stated that they were already utilizing similar methods to MI or that patient resistance to change hindered progress. One CHA said that the patients “try to follow the plans and some succeed. Some cases are more difficult”. Two CHAs remarked on the increased time required to implement these techniques, although this was not necessarily described as a negative change by one CHA: “We turned more into listeners than talkers. Sometimes when I go on the visit it would end up taking an hour and a half!”

When asked if they will continue to use MI methods and action planning in their work, nearly all CHAs said they would incorporate these methods into their work in some capacity. Some CHAs described how these methods can be used beyond diabetes management for whatever the patient’s health priorities are and noted ways they plan to adapt the methods to better fit their day-to-day work, including using a “milder” form of the intervention. One CHA emphasized the versatility of these methods, stating she can use them “in any situation, even in my house. I can use them in my life.” Other CHAs also described their intention to use the methods on themselves, with another CHA stating that she has already made herself an action plan for healthy behavior changes she wanted to make.

## Discussion

All but three of the trained community health agents (CHAs) employed at a public primary care center serving a low-income urban community in the city of São Paulo, Brazil achieved proficiency in basic Motivational Interviewing (MI) approaches to support diabetes patients’ self-management as part of routine service delivery in their mandatory monthly home visits. Diabetes patients with poor glycemic control who received home visits from trained CHAs over 6 months reported significant improvements in their satisfaction with the diabetes care they received and in key dimensions of their interactions with their CHAs. They also reported improvements in physical activity, consumption of fruits and vegetables, and adherence to diabetes medications, all important diabetes self-management behaviors. Overall, most CHAs interviewed spoke very positively about the MI skills they learned, reported that their communication with patients had improved, and planned to continue incorporating at least some of the skills they learned in their future interactions with patients.

Prior studies conducted in the United States have found favorable outcomes among adults with chronic conditions such as diabetes and at high risk for diabetes and coronary heart disease from programs led by community health workers trained in evidence-based counseling approaches [23, 26–29, 44]. To date, however, most prior studies have evaluated stand-alone programs led by trained lay health workers. This pilot study is one of the few studies that has examined efforts to improve the counseling skills of lay health workers in routine service delivery in an LMIC setting in which the workers have multiple other responsibilities and often less formal education and training than their counterparts in high income countries. A study by Dewing et al. [43] analyzed the ability of lay health workers to deliver an evidence-based sexual risk reduction intervention in South Africa using MI techniques. Similar to our study, these workers received both initial and follow-up booster training (35 h initial training and 18 h of follow-up booster training and supervision over a 12-month period). A prior study of theirs had found that the lay health workers did not achieve proficiency in MI skills with just initial training. However, adding ongoing training and feedback improved their therapeutic approach and communication skills and enabled the delivery of higher quality health counseling. In that study, the lay health workers did not achieve proficiency in more advanced MI skills (such as formal readiness-to-change assessments) that we did not attempt to teach CHAs in our study. As in our study, the lay health workers trained in Dewing et al.’s intervention successfully increased the extent to which they actively elicited patients’ goals, engaged in active listening, and displayed respect and empathy for patients.

Our study findings support the value of conducting a larger-scale evaluation comparing outcomes between UBS’s in which CHAs receive behavioral counseling training and ongoing booster training and feedback and UBS’s in which CHAs do not receive such training. In light of the multiple competing priorities and demands facing health care workers in primary care centers in low-income communities in Brazil, such a rigorous evaluation that includes cost-effectiveness data is an important next step to inform whether this initiative would merit allocation of scarce resources to be implemented more widely. In light of reports from some CHAs about the additional time they spent in home visits to set and follow up on diabetes self-management goals, further attention to this concern will also be necessary in future interventions. Moreover, while diabetes is a particularly high-cost condition and is thus considered a public health priority, the approach evaluated in this pilot could and should be used with adults facing a range of health challenges that require initiation and maintenance of healthy behaviors. Finally, there is much to be learned about the most effective approaches to train and provide ongoing support to community health workers and other health workers to provide effective behavioral counseling in face-to-face encounters as well as through other modalities such as text messages [58].

This pilot study must be interpreted in the face of several limitations. Most significant, it is a single- group, pre-post study with no control group, which is a weak study design that does not account for secular trends, regression to the mean, or other factors that might have led to the observed changes over the study period. Of note, two such factors over the study period may have contributed to worsening diabetes risk factor control among the participants: 1) half of the physicians employed at the center left during the intervention period and were not yet replaced, creating significant access barriers for patients; and 2) there was a 2-month period during which there were no sulfonyurea medications at the center or regional pharmacies leading to 19 participants being off these medications. Thus, the improvements found in patients’ assessments of the quality of their diabetes care and in measured clinical values of A1c, lipids, and triglycerides occurred in spite of these disruptions to diabetes care at the study health care center. Our evaluation therefore may have underestimated the potential efficacy of the intervention. A second important limitation is that because the intervention was only conducted at one site, our findings may not generalize to other primary care centers. Finally, we did not measure patient characteristics such as co-morbidities and number of medications that would influence changes in outcomes such as medication adherence.

## Conclusion

The limitations notwithstanding, this pilot study supports the feasibility and acceptability of training primary care center-based community health agents (CHAs) in Motivational Interviewing-based approaches to improve adults’ self-management of their diabetes during routine monthly home visits. Moreover, we found promising improvements in participants’ assessments of the quality of diabetes care they received and of their interactions with their CHA as well as in key diabetes self-management behaviors and clinical risk factors. In resource-scarce settings, it is critically important to fully utilize existing primary care resources to stem the growing epidemic of diabetes and other NCDs. Brazil has been a leader in recognizing the potential value of CHAs by incorporating them fully into primary care centers’ health care teams. To inform efforts to fully meet this potential, we need to develop and rigorously evaluate over a longer time period larger-scale CHA-led programs similar to the one tested in this pilot.

## Abbreviations

CHA: Community health agents
HCCQ: Health Care Climate Questionnaire
LMIC: Low and middle income country
MI: Motivational interviewing
NCDs: Noncommunicable diseases
PACIC: Patient Assessment of Chronic Illness Care scale
UBS: *Unidade Básica de Sáude* (Basic Health Unit)
